# Genome-wide differential DNA methylation analysis of MDA-MB-231 breast cancer cells treated with curcumin derivatives, ST08 and ST09

**DOI:** 10.1186/s12864-022-09041-2

**Published:** 2022-12-06

**Authors:** Snehal Nirgude, Sagar Desai, Bibha Choudhary

**Affiliations:** 1 Institute of Bioinformatics and Applied Biotechnology, Electronic city phase 1, 560100 Bangalore, India; 2 Working at Division of Human Genetics, Children’s Hospital of Philadelphia, 19104 Philadelphia, PA USA

**Keywords:** Curcumin derivatives, Differential methylation, Whole genome bisulfite sequencing (WGBS), integrated approaches, drug-specific response, triple-negative breast cancer (TNBC)

## Abstract

ST08 and ST09 are potent curcumin derivatives with antiproliferative, apoptotic, and migrastatic properties. Both ST08 and ST09 exhibit in vitro and in vivo anticancer properties. As reported earlier, these derivatives were highly cytotoxic towards MDA-MB-231 triple-negative breast cancer cells with IC50 values in the nanomolar (40-80nM) range.In this study,we performed whole-genome bisulfite sequencing(WGBS) of untreated (control), ST08 and ST09 (treated) triple-negative breast cancer cell line MDA-MB-231 to unravel epigenetic changes induced by the drug. We identified differentially methylated sites (DMSs) enriched in promoter regions across the genome. Analysis of the CpG island promoter methylation identified 12 genes common to both drugs, and 50% of them are known to be methylated in patient samples that were hypomethylated by drugs belonging to the homeobox family transcription factors.Methylation analysis of the gene body revealed 910 and 952 genes to be hypermethylatedin ST08 and ST09 treated MDA-MB-231 cells respectively. Correlation of the gene body hypermethylation with expression revealed CACNAH1 to be upregulated in ST08 treatment and CDH23 upregulation in ST09.Further, integrated analysis of the WGBS with RNA-seq identified uniquely altered pathways - ST08 altered ECM pathway, and ST09 cell cycle, indicating drug-specific signatures.

**Supplementary Information:**

The online version contains supplementary material available at 10.1186/s12864-022-09041-2.

## Introduction

The cancer genome is characterised by global hypomethylation and promoter hypermethylation [1–5]. DNA methylation occurs at the carbon 5th position of cytosines(5meC) and plays a crucial role in gene regulation. Hypomethylation leads to unwanted transcription of repeat elements, abnormal activation of individual genes, disruption of chromosome replication control leading to genomic instability and reactivation of transposons. Promoter localized hypermethylation can lead to aberrant silencing of genes involved in developmental transcription factors, tissue remodelling genes, DNA repair genes, cell cycle control genes, anti-apoptotic genes, metastatic genes, anti-angiogenic genes, also classified as tumour suppressor genes that exhibit anticancer properties [1, 5, 6].

Bisulfite sequencing (BS) to analyse DNA methylation has been a gold standard method since its first use in 1992 [7]. However, the coupling of BS treatment with next-generation sequencing (NGS) has resulted in understanding the DNA methylation pattern at a whole-genome (WGBS) scale [8]. WGBS is the only method to obtain information about % methylation at a single CpG site resolution and study genome-wide DNA methylation [9].Hence, WGBS is the standard profiling method that is widely incorporated by major epigenome consortiums such as NIH Roadmap [10], ENCODE [11], Blueprint [12], and IHEC [13]. Methylation patterns generated via WGBS can be used to develop biomarkers specific for tumour type, markers for risk assessment, early detection and monitoring of prognosis, and indicators of susceptibility or response to therapy [4]. Bioactive food components like folate, polyphenols, selenium, retinoids, fatty acids, isothiocyanates, and allyl compounds influence epigenetic processes via DNA methylation [6].

Triple negative breast cancer (TNBC) is the most aggressive breast cancer [14]. Since TNBC tumours do not express estrogen receptor (ER), progesterone (PR), or human epidermal growth factor receptor (HER2), TNBC patients do not benefit from endocrine therapy or trastuzumab [14]. Thus, alternative strategies need to be developed. Since epigenetic dysregulation is an early step in carcinogenesis and is reversible, intervention strategies can target and modify the cancer epigenome [15]. Normal DNA methylation patterns on oncogenes and tumour suppressor genes can be restored and proposed for cancer prevention [5, 6, 15, 16]. Drugs like 5-azacytidine, a cytidine analogue, and 5-aza-2′-deoxycytidine have been tested to reactivate gene expression silenced by hypermethylation, reducing the malignant cell burden and improving patient survival [17, 18]. However, 5-azacytidine induces non-specific global methylation changes. On the other hand, curcumin is reported to cause methylation changes only in a subset of partially-methylated genes [19].

Epigenetics is the study of heritable changes in gene expression that occur independently of changes in the primary DNA sequence [20]. DNA methylation, covalent histone modifications, nucleosome positioning and miRNAs are the major players in epigenetic mechanisms.Our lab has extensively explored the global epitranscriptome (miRNA and mRNA) of cancer cells induced by Curcumin [21] and its derivatives ST08 [22] and ST09 [23]. We performed a comprehensive, systematic whole genome DNA methylation analysis to understand whether the drugs ST08 and ST09 regulate gene expression via DNA methylation. We investigated the impact of curcumin (a polyphenol) derivatives - ST08 and ST09, on DNA methylation of MDA-MB-231 breast cancer cells (TNBC cell line).

## Materials and methods

### Cell Culture

MDA-MB-231 cells were purchased from the National Centre of Cell Culture (NCCS), Pune, Maharashtra, India and were grown in Dulbecco’s Modified Eagles Medium (DMEM high glucose with L-glutamine; Lonza). The media was supplemented with heat-inactivated 10% fetal bovine serum (Gibco), 100 IU mg/mL penicillin/streptomycin (Gibco) and cells were maintained at 37 °C in a humidified atmosphere containing 5% CO2. 100 mM stocks of ST08 and ST09 were prepared in DMSO, and all the treatments had equal concentrations of dimethyl sulfoxide (DMSO) between 0.1 and 0.2%.

### Genomic DNA isolation

1.0 × 10^5 cells were seeded in each well of a 6-well plate. After 24 h cells, MDA-MB-231 cells were treated with 75nM ST08 and 50nM ST09. After 48 h of treatment, cells from three wells having the same treatment were pooled together by trypsinization and washed with PBS twice. DNA isolation was performed using a DNA lysis buffer( 100mM NaCl, 1% SDS, 0.1% Triton X-100, 10mM EDTA, 100mM Tris-HCl pH8, 50ug/ml Proteinase K). The lysed cells were then subjected to phenol-chloroform extraction, and DNA was precipitated using isopropanol and washed with 70% ethanol. DNA samples were dissolved in the TE buffer.

### Whole genome Bisulfite Illumina library preparation and sequencing

5 µg of genomic DNA isolated from treated MDA-MB-231 cells were used as input for library preparation. 260/280 ratio for each sample was calculated, and samples with ratios of 1.8–2.0 were considered. Covaris shearing was used to generate dsDNA fragments with 3ʹ or 5ʹ overhangs of 250 bp (peak size). Bisulfite treatment was given to convert any unmethylated Cytosine to Thymidine. End repair using T4 DNA polymerase and Klenow enzyme was performed to repair overhangs. 3’ ends were adenylated to prevent them from ligating one another during the adapter ligation reaction. After methyl-adapter ligation, DNA fragments were enriched using PCR [24]. After constructing the libraries, their concentrations and insert sizes were detected using Qubit and Agilent Tapestation, respectively. High throughput sequencing was performed using Illumina HiSeq2500 to obtain 100-bp paired-end reads. The WGBS for ST08 and ST09 treatments and transcriptome data for ST08 treatment are available at PRJNA794262. The WGBS read summary is detailed in Supplementary Table 1.

### Differential methylation

**Fig. 1:**
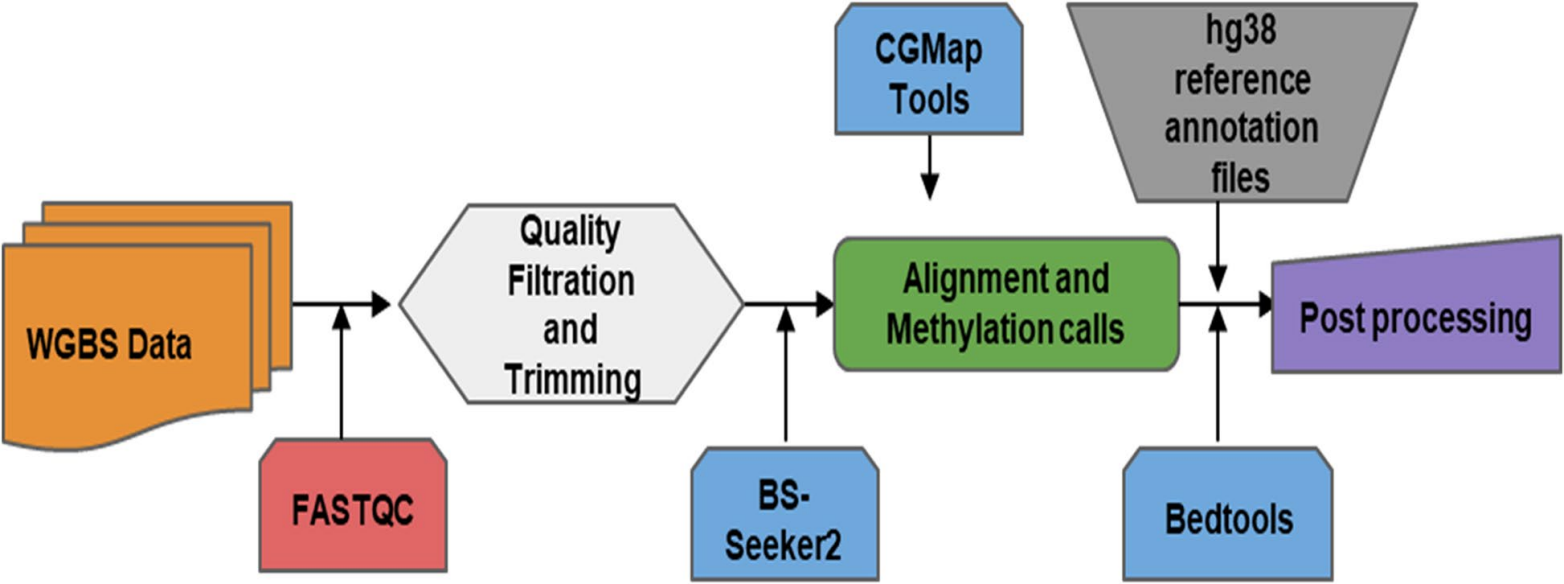
Pipeline for WGBS data analysis

Raw bisulfite sequencing reads were checked for quality using FastQC [25], and adapter trimming was performed using trim_galore [26]. The human hg38 reference genome was prepared for bisulfite analysis, followed by filtered reads’ alignment to the same was performed using the BS-Seeker2 tool [27]. In the CG context, methylated sites were extracted using CGmapTools [28] for control and the treated samples (Fig. 1).

Using CGmapTools [28], significant differential methylation sites were obtained between Control and ST08 and Control and ST09. CpG sites with a difference of greater than or equal to 0.75 ∆β-value were termed Hypermethylated, and less than those with a difference of less than 0.25 ∆β-value were termed Hypomethylated. Having obtained the differentially methylated sites, Hyper and Hypo methylated promoters were segregated by intersecting the human hg38 promoter bed file from UCSC [28, 29] genome browser, differential methylation sites using the BEDTools [30] suite. The differentially methylated promoters were compared to the corresponding differentially expressed genes from transcriptome analysis. The data was segregated into 2 two categories; (1) hypermethylated promoters - downregulated genes, and (2) hypomethylated promoters - upregulated genes. The resulting genes were further categorised into Tumor suppressors and oncogenes with the Tumor Suppressor Gene database [31] and the Oncogene database [32]. Candidate genes for analysis were compared with the differential methylation statuses from TCGA [33] data using the online Smart App [34]. The final set of genes was  reported, and a bar graph of methylation level vs. the log2FC from transcriptome analysis was plotted. All the plots were generated using Microsoft Excel, and the significance of differential methylation was performed by CGmapTools [28] using the Chi-square test.

Further, the differentially methylated gene bodies were obtained by intersecting the human hg38 gene body annotation bed file from the UCSC genome browser with the differentially methylated sites. The data was segregated into 2 two categories; (1) hypermethylated gene bodies and (2) hypomethylated gene bodies; finally, the overexpressed genes with hypermethylated gene bodies.

### Validation of the methylation states using SMARTapp

To check if the drug-induced changes in the genes correlated with alterations in the methylation status of the genes known to be differentially methylated in normal/tumour breast tissue samples, we used publicly available breast cancer methylation data through the SMARTapp database. The SMART (Shiny Methylation Analysis Resource Tool) App is a web application for comprehensively analysing the DNA methylation data of The Cancer Genome Atlas(TCGA) project [35]. It facilitates the integration of multi-omics and clinical data with DNA methylation. It provides key interactive and customised functions, including CpG visualisation, pan-cancer methylation profile, differential methylation analysis, correlation analysis and survival analysis for users to analyse the DNA methylation in diverse cancer types multi-dimensional.

### Statistical analyses

All the plots were generated using Microsoft Excel, and the significance of differential methylation was performed by CGmapTools [28]using the Chi-square test.The SMART App performs correlation analysis between gene expression and methylation for any given sets of TCGA, using methods including Pearson, Spearman, and Kendall correlation statistics [35].The differentially expressed genes from transcriptome analysis were analysed using a hypergeometric test and the Benjamini & Hochberg method [22].

## Results

The total number of C’s evaluated was 36,966,450 (ST08), 36,256,297(ST09), and 339,121,709(control), and methylated C’s in CpG analysed accounted for 59.6%, 58.5%, and 54.7% in ST08, ST09, and control MDA-MB-231 cells respectively (Supplementary Fig. 1). Further analysis of methylated Cs in the CpG island context revealed the highest methylation in control (5.8%) vs. ST08 (5.24%) and ST09 (5.37%), indicating hypomethylation at CpG islands induced by ST08 and ST09 in MDA-MB-231 cells (Supplementary Fig. 1).

### ST08 and ST09 altered methylation patterns in MDA-MB-231 cells in a drug-specific manner

**Fig. 2:**
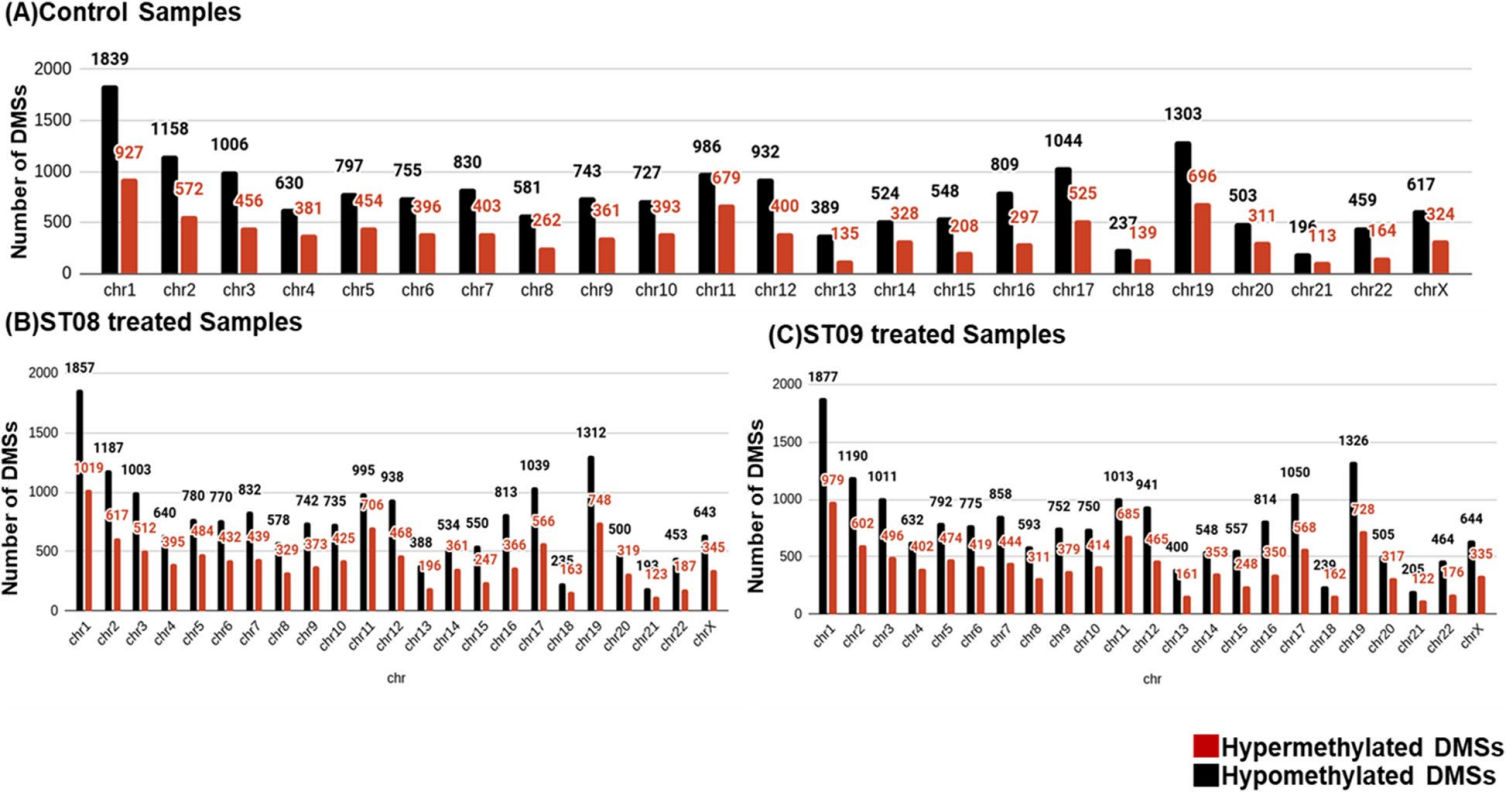
Distribution of hypermethylated and hypomethylated Differentially methylated sites (DMSs) along each chromosome. MDA-MB-231 control cells (**A**), treated with ST08 (75 nM) (**B**), ST09 (50nM) (**C**) for 48 h. Numbers up the bars indicate the number of genes according to their position in chromosomes. The red bar indicates hyper, and the black bar indicates hypomethylation

To begin with methylation status analysis, all the differentially methylated CpG sites (DMSs) across the genome were evaluated. DMS with beta value > 0.75 (hypermethylated) and < 0.25 (hypomethylated) were analyzed. To understand if there was a chromosome bias in the methylation status, we plotted all the methylated sites identified by CgmapTools (Fig. 2). All the chromosomes showed hypomethylation compared to control and lower hypermethylation in chromosomes 3, 5, 8,17, and 22 in ST08 treated cells. Only chromosome 5 showed lower hypermethylation in ST09 treatment, whereas overall hypomethylation was similar to ST08. Further, to understand if the methylated sites were differentially distributed in the gene body or the promoter region, we performed the methylated CG site analysis of the gene body. Differentially methylated gene bodies were obtained by intersecting the human hg38 gene body annotation bed file from the UCSC genome browser with the differentially methylated sites in MBA-MB-231 treated with ST08 and ST09. We obtained 51 hypomethylated gene bodies in ST08 treated samples and 37 in the case of ST09. 910 and 952 hypermethylated gene bodies were obtained in ST08, and ST09 treated samples, respectively. Further investigating the gene bodies for a chromosomal bias, we observed Chr 19 with the most hypermethylated sites in both treatments. To check if the hypermethylated gene bodies correlate with high expression of the genes, we intersected hypermethylated gene bodies with overexpressed genes, and surprisingly, we found only 2 genes, CACNA1H and RPL31, in the ST08 treated sample and 2 genes, KCNN1, CDH23 in the case of ST09 treated sample. CACNA1H is a voltage gated calcium channel whose expression was found to positively correlate with a decrease in brain metastasis in the breast cancer PDX model [36]. Similarly, in ST09 treatment, the gene hypermethylated and overexpressed was CDH23, cadherin 23, and mutations in this gene have been found in younger breast cancer patients [37].

For analysing the level of methylation in the promoters of the genes, 38,000 promoters were extracted, of which ~ 27,000 CpG were analysed. DMS with beta value > 0.75 (hypermethylated) and < 0.25 (hypomethylated) were analyzed. In control MDA-MB-231 cells, 69% of the CpGs in promoters showed a differential where 66% of promoters were hypermethylated and 33% hypomethylated. ST08 treatment led to a decrease in hypermethylation to 64.3% and an increase in hypomethylation to 35.6%. A similar trend was also observed in ST09 treated cells, where a decrease in hypermethylation to 65% and an increase in hypomethylation to 35%, indicating drug-induced hypomethylation. This analysis indicated widespread hypomethylation, yet drug-specific change in the number of promoters is evident.

Further analysis was carried out using CpGs in the CpG island context.

### Chromosome 9 methylation identifies ST08 and ST09 specific signatures in MDA-MB-231 cells

**Fig. 3:**
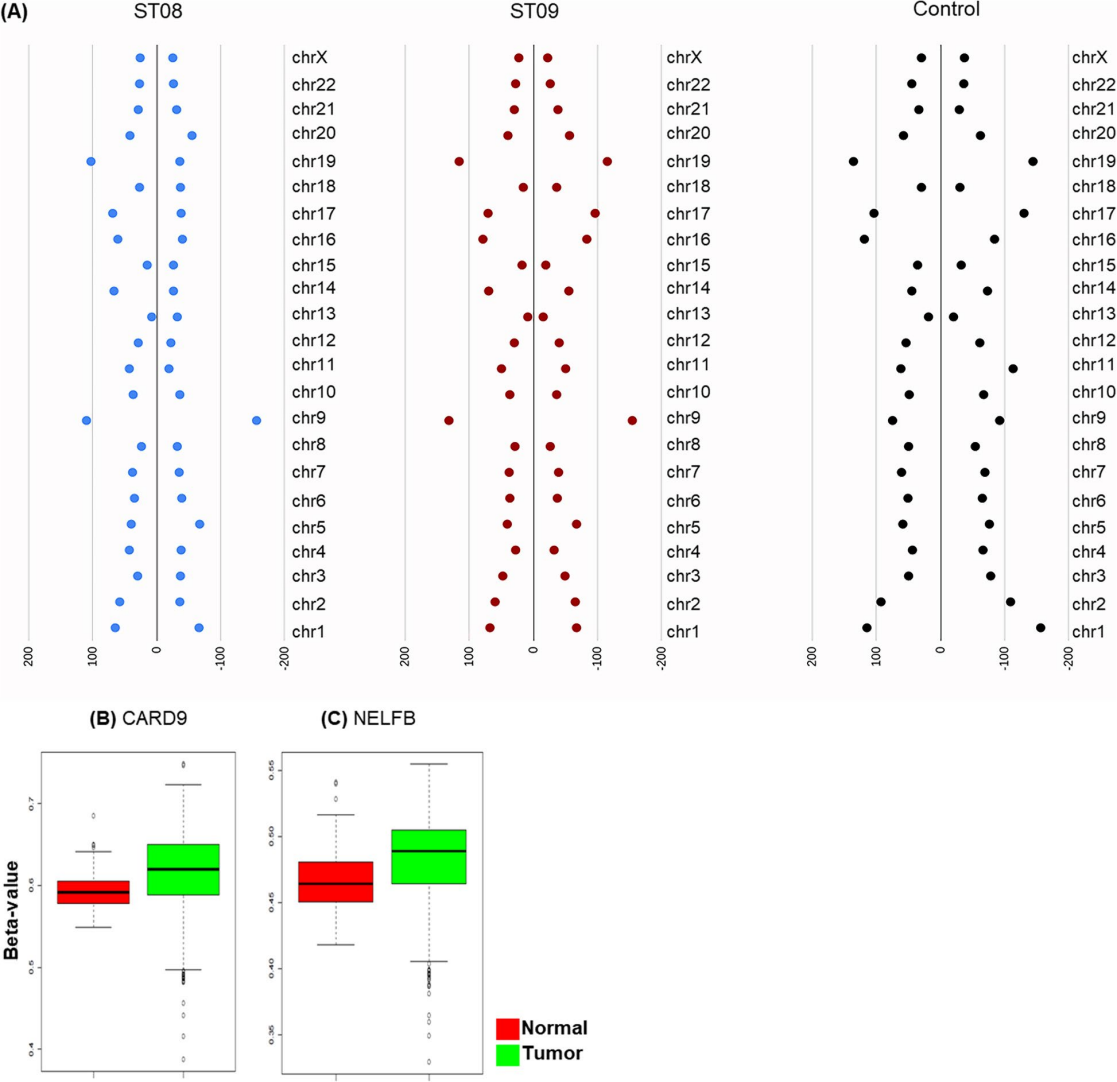
**A** Distribution of hypermethylated and hypomethylatedDifferentially methylated regions (DMRs) along each chromosome. Promoter methylation status of genes in normal and tumour breast cancer patient samples using SMART App [34]. **B **CARD9, **C **NELFB X-axis represents Δβ-value and Y-axis represents normal and tumor samples

To understand the distribution of methylated CpGs in the context of CpGisland, we analysed CpG island in the promoter. In MDA-MB-231 cells, unlike the DMS, CpG islands were hypomethylated in control MDA-MB-231 cells. Chromosomal level methylation analysis at CpGsrevealed Chr9 to have the most altered CpGs; both hypermethylation and hypomethylatedCpGs were abundant in ST08, and ST09 treated MDA-MB-231 cells (Fig. 3A**)**. Unlike DMS specific to the drug, both drugs showed a similar altered methylation pattern at CpG islands on chromosome 9. To determine whether both drugs altered the same CpG sites, hypomethylated and hypermethylated genes on chromosome 9 were collated. Interestingly, 10% of the genes on chromosome 9 showed differential methylation, of which 32 were common to both treatments. 13 genes had CpGshypomethylated in ST09 and ST08 treated MDA-MB-231. Only 4 genes showed hypermethylation, indicating hypomethylation of CpG. Common hypomethylated genes were *CARD9, NDOR1, NELFB, PTGES, MAN1B1, PMPCA, RNU6ATAC, DCTN3, ADGRD2, LRRC26*. To check whether the genes which showed hypomethylation upon drug treatment were hypo or hypermethylated in breast cancer patients, we checked for the methylation status of the genes and found CARD9 and NELFB to be hypermethylated in breast cancer samples using SMARTapp [34] (Fig. 3B, C).

For the genes which showed both hyper and hypomethylatedCpG sites on the CpGisland on chromosome 9, we checked for the expression levels of the transcripts.

### Functional characterisation of the CpG island associated genes on chromosome 9 using RNA-seq

**Fig. 4:**
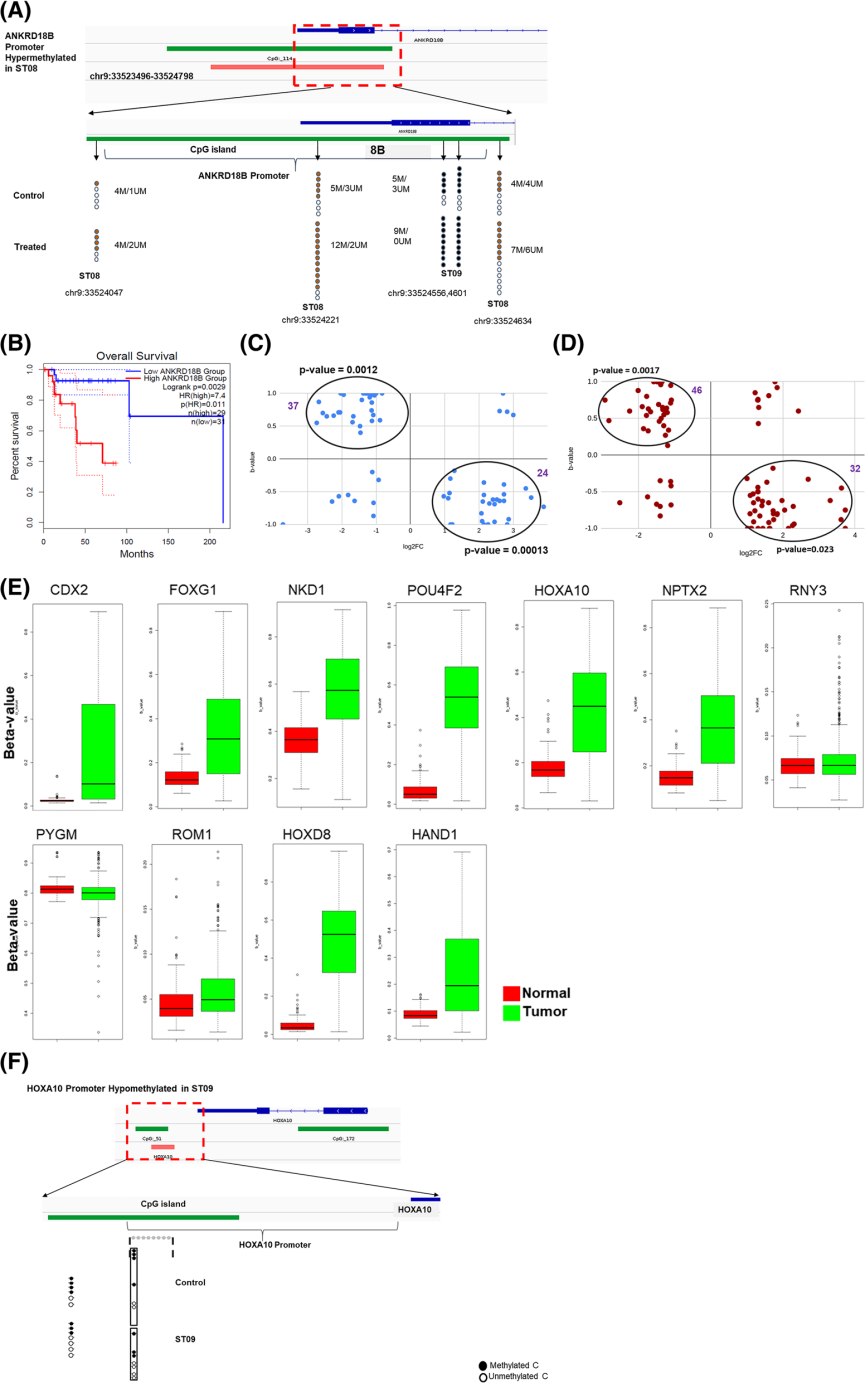
**A **Hypermethylation of ANKRD18B promoter by ST09 treatment. **B** Overall survival of breast cancer patients shown by GEPIA analysis of ANKRD18B.Changes in ST08, ST09-mediated modification of DNA methylation in CpG island correlate with changes in gene expression. We performed genome-wide gene expression analyses to evaluate if ST08, ST09-mediated DNA methylation changes correlated with gene expression variation. Genes that showed reproducible DNA methylation changes after ST08 (**C)**, ST09 (**D**) treatment (Δβ > 0.1) were matched with genes that showed ≥ 1-fold differences in gene expression. Those genes that showed an inverse correlation between methylation and gene expression changes are encircled in the graph. **E** Promoter methylation status of genes in normal and tumor breast cancer patient samples using SMART App [34]. **F** hypomethylation of HOXA10 promoter by ST09 treatment

To check whether the genes which were hypomethylated/hypermethylated were also expressed, RNA seq was performed upon ST08 [22] and ST09 treatment (Additional file 2). All the differentially expressed genes with log2FC > 1 and < 1 were analysed for overlap with 32 genes. Among 32 genes that showed hypo and hypermethylatedCpG sites on CGIs on chromosome 9, only 1 gene common to ST08 and ST09 was downregulated, indicating the influence of hypermethylatedCpG. The sites methylated differentially are distinct Cs at a specific position. For example, Fig. 4A shows hypermethylation of *ANKRD18B *CpG sites post ST09 treatment. The gene whose expression was significantly altered was *ANKRD18B*. *ANKRD18B* was downregulated upon ST08 and ST09 treatment. We checked the expression of *ANKRD18B* in breast cancer patients using GEPIA and found it to be upregulated. Interestingly, survival analysis showed a significant association with low survival in Her2 + ve breast cancer (Fig. 4B).

### Functional characterisation of the CpGisland associated genes across the genome using RNA-seq

Further, we analysed all CGIs which were hypomethylated and hypermethylated at CpGisland in the promoter region and correlated with expression. (33 out of 98 genes,are downregulated; hypergeometric test; FC 2; *P*-value < 0.001) and are methylated, whereas depleted in methylation is observed in upregulated genes (44 out of 98 genes are upregulated; hypergeometric test; FC 2; *P*-value < 0.05) in ST09 treated cells. (29 out of 82 genes,are downregulated; hypergeometric test; FC 2; *P*-value < 0.001) and methylated, whereas, CpGislands depleted in methylation is observed in upregulated genes (25 out of 82 genes are upregulated; hypergeometric test; FC 2; *P*-value < 0.05) in ST08 treated MDA-MB-231 cells. (Fig. 4C, D). The upregulated genes on functional annotation using KEGG [38] returned DNA-BINDING’, ‘TRANSCRIPTION,‘ ‘TRANSCRIPTION REGULATION,‘ ‘HOMEOBOX.‘ Most of the genes were transcription factors such as *BARHL2, CDX2, POU4F2, HOXA10, HAND1, and FOXG1*. We used SMARTApp to check the status of these transcription factors in Breast Cancer samples compared to control. Interestingly, all of the genes were hypermethylated in Breast cancer, and the drugs ST08 and ST09 induced hypomethylation correlated with high expression in MDA-MB-231 cells (Fig. 4E). We further plotted single CpG sites within CpG islands using CGmap Tools [28] and found that CpG sites on the HOXA10 promoter region in ST09 treatment showed hypomethylation at multiple CpG sites (Fig. 4F).

Having analysed CpG islands, we then checked for the differentially methylated sites and their correlation with expression and identified drug-specific pathways.

### ST08, ST09-induced DNA methylation alterations Associated with corresponding changes in Gene expression

**Fig. 5:**
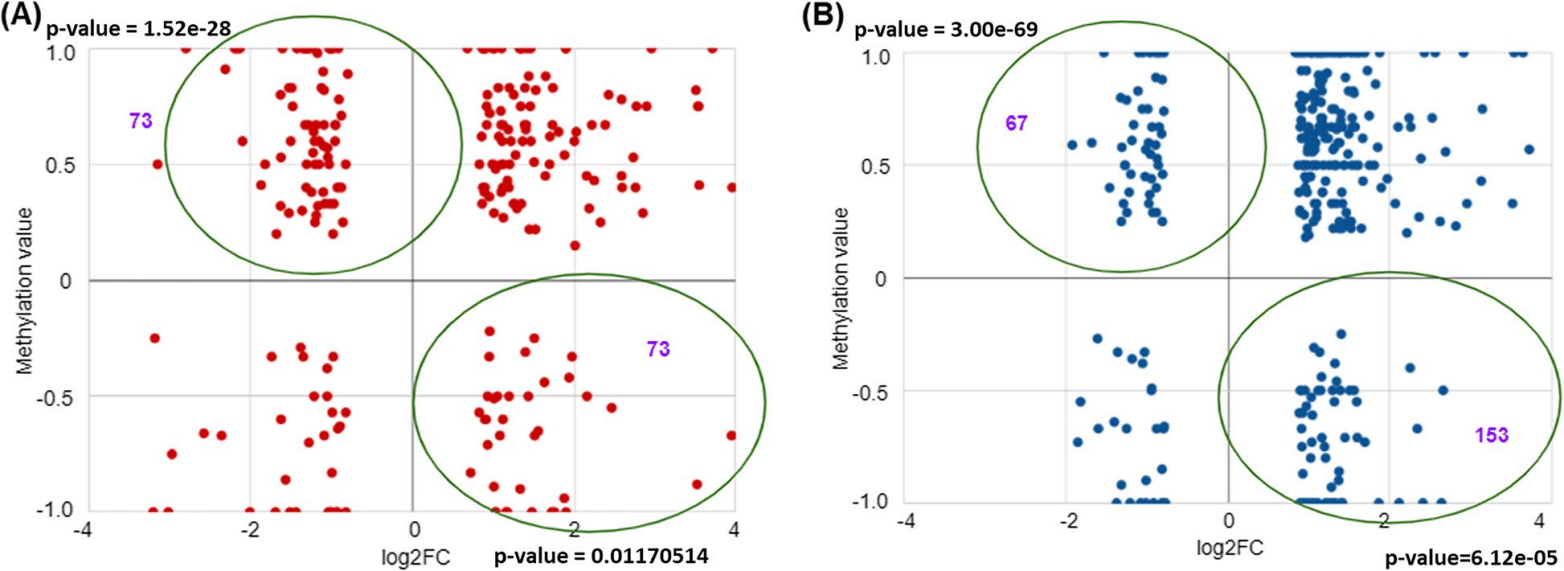
Changes in ST08 and ST09-mediated DNA methylation modification correlate with gene expression changes. We performed genome-wide gene expression analyses to evaluate if ST08 and ST09-mediated DNA methylation changes correlated with gene expression variation. Genes that showed reproducible DNA methylation changes after ST08 (**A)**, and ST09 (**B**) treatment (Δβ > 0.1) were matched with genes that showed ≥ 1-fold differences in gene expression. Those genes that showed an inverse correlation between methylation and gene expression changes are encircled in the graph

To understand the biological significance of DNA methylation changes induced by ST08 and ST09, we performed a correlative analysis between genome-wide methylation results and the gene expression data. Herein, we selected all CpG loci ( CG islands, CG shores and CG sites) in the promoter of genes with a methylation change of at least 10% (or a Δβ-value ± 0.1) and an expression change of at least log_2_fold > 0.5 between control and ST08, ST09 treated cells. We identified 73 (73/241, FC 2, hypergeometric test, *p* < < 0.001) hypermethylated promoters in ST08 and 67 ( 67/346. FC 2, hypergeometric test, *p* < < 0.001) in ST09 treated MDA-MB-231 cells using these criteria. The expression of the corresponding transcripts was downregulated (Fig. 5). Similarly, 73 (73/241, FC 2, hypergeometric test, *p* < 0.01) and 153 genes (153/346, FC 2, hypergeometric test, *p* < < 0.001) in ST08, ST09 treated cells, respectively, had hypomethylated promoters, and corresponding transcripts were upregulated (Fig. 5).

We subjected the genes obtained to tumor suppressor genes (TSG) and oncogenes gene analysis.

### Tumor suppressor and oncogene analysis

**Fig. 6:**
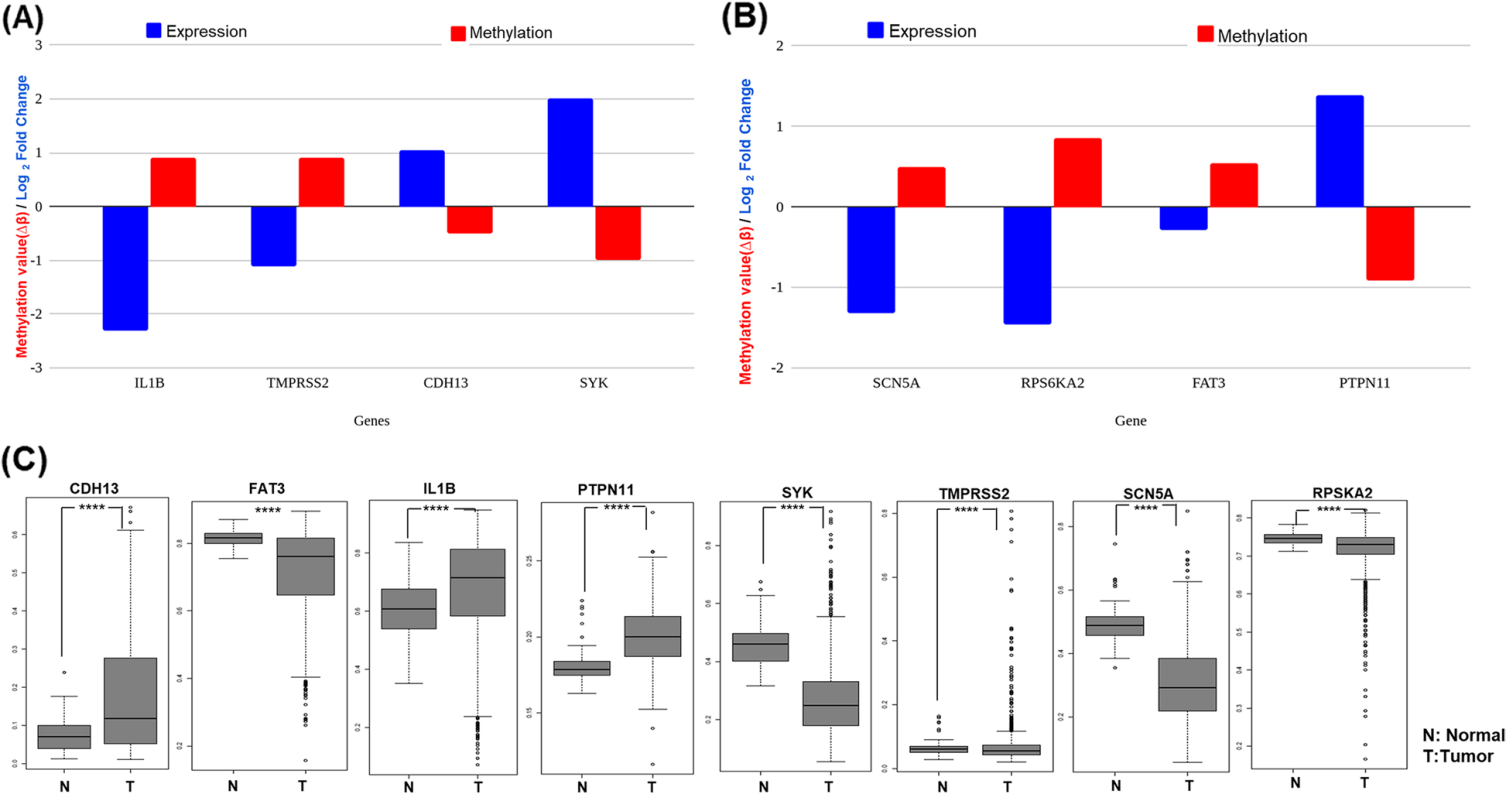
Bar graph showing inverse correlation between methylation and gene expression status for ST08 (**A**) and ST09 (**B**) treatment in MDA-MB-231 cells. **C** Promoter methylation status of genes in normal and tumor breast cancer patient samples using SMART App [34]. X-axis represents Δβ-value and Y-axis represents normal, tumor sample.(ns: *p* > 0.05; *: *p* < = 0.05; **: *p* < = 0.01; ***: *p* < = 0.001; ****: *p* < = 0.0001)

Cancer is driven by an imbalance of oncogene gene and tumor suppressor gene expression. To find the TSGs and oncogenes regulated by promoter methylation, we subjected downregulated genes with hypermethylated promoter status for oncogene analysis and upregulated genes with hypomethylated promoter status TSG analysis. For TSG analysis, we used the TSG database [39] specific for breast adenocarcinoma, and for oncogene analysis, we used the Oncogene database [40]. The percentage of upregulated TSGs and downregulated oncogenes were calculated using the genes with expressions correlating with DNA methylation (Table 1). Table 2 gives the list of TSGs and Oncogenes regulated by both drugs. *IL1B* and *TMPRSS2* were two commonly hypermethylated oncogenes downregulated by ST08 and ST09. Overall, DNA methylation led to similar changes in TSG and oncogene expression. Few of these genes were plotted as a bar graph, which shows the opposite correlation between gene expression and promoter methylation status. ST08 led to hypermethylation of oncogenes like IL1B, TMPRSS2 leading to their downregulation and hypomethylation of TSGs *CDH13, SYK* leading to their upregulation (Fig. 6A). ST09 led to promoter hypermethylation and downregulation of oncogenes SCN5A, RPS6KA2, and promoter hypomethylation and upregulation of TSGs *FAT3, PTPN11* (Fig. 6B). We also checked the methylation status of these genes in breast cancer patient data from The Cancer Genome Atlas (TCGA) using the SMART App [34]. The promoter analysis of *IL1B, CDH13*, and *PTPN11*, using SMART App, showed methylation status in breast tumor patient samples **(**Fig. 6C**)**. Interestingly, CDH13 (TSG) is hypermethylated in breast cancer patients, and the drug ST08 induced hypomethylation and tumor suppressor expression.

**Table 1:** % of TSGs upregulated by promoter hypomethylation and % of Oncogenes downregulated by promoter hypermethylation after each treatment

	ST08	ST09
**TSGs(% Upregulation)**	11	9
**Oncogenes(% downregulation)**	16	12

**Table 2:** List of TSGs and Oncogenes regulated by ST08 and ST09 via DNA methylation

ST08 mediated hypomethylated and upregulated TSGs	*HNF4A,PGR,SYK,CDH13,DAPK1,SPARC,THY1,PTPRT*
ST08 mediated hypermethylated and downregulated Oncogenes	*LAMA3,IL1B,NR3C2,UACA,SIRPA,TMPRSS2,PLB1,CHD1,GLIPR2,TXNIP,CCDC28A,DNMT3A*
ST09 mediated hypomethylated and upregulated TSGs	*AIM2,ATM,BRCA1,CUL2,CUL5,HSPD1,IQGAP2,LRMP,MLH3,NBN,NRCAM,PTPN11,RBBP8,TOPORS*
ST09 mediated hypermethylated and downregulated Oncogenes	*ACOXL,CSMD3,FAT3,IL1B,SCN5A,SRPX,SYN2,TMPRSS2*

### Correlating ST08 regulated ECM pathway and ST09 cell cycle in MDA-MB-231 with promoter hypermethylation

**Fig. 7:**
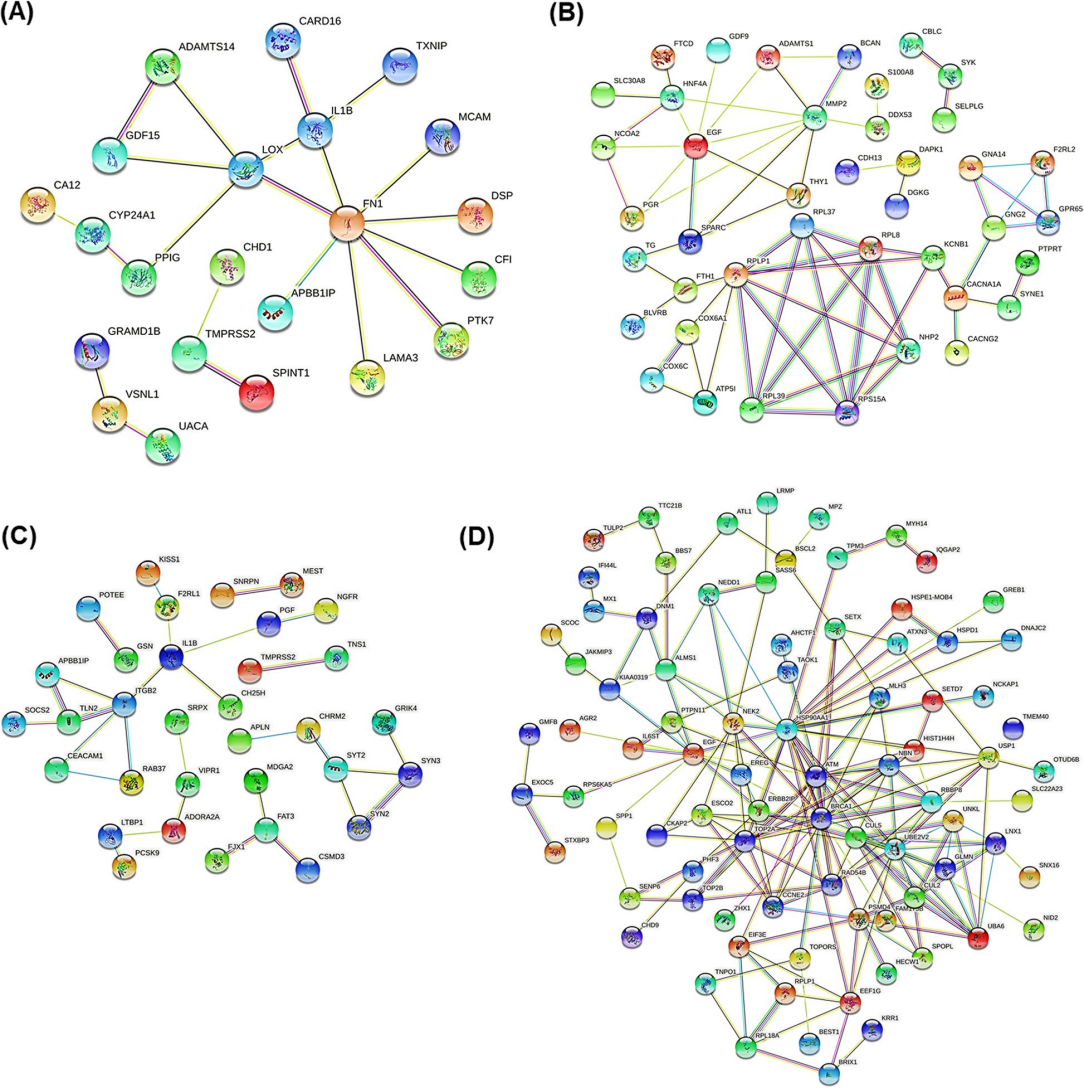
Protein-protein interaction(PPI) network generated for correlated genes using STRING database (**A**) PPI network of genes with hypermethylated promoters upon ST08 treatment in MDA-MB-231 cells, (**B**) PPI network of genes with hypomethylated promoters upon ST08 treatment in MDA-MB-231 cells, (**C**) PPI network of genes with hypermethylated promoters upon ST09 treatment in MDA-MB-231 cells, (**D**) PPI network of genes with hypomethylated promoters upon ST09 treatment in MDA-MB-231 cells

The genes with an inverse correlation between promoter methylation and fold change were further subjected to network and pathway analysis using the STRING database [41]. We got four intricate networks, two for ST08 and ST09 (Fig. 7). We found that ST08 downregulated the pathways related to extracellular matrix (ECM) by downregulating the genes in the pathway [22, 42] by promoter hypermethylation. ST08 upregulated ribosome-related GO term by upregulating genes in the pathway by promoter hypomethylation. Similarly, ST09 downregulated the cell-cell signaling pathway and upregulated the DNA double-strand break repair pathway [43]. (Additional file 2 provides the list of genes for generating the network).

## Discussion

The study focussed on novel epigenetic data highlighting the relevance of ST08 and ST09 on cancer. Triple-negative breast cancer tests negative for estrogen receptors, progesterone receptors, and the absence of HER2 protein overexpression [44]. It accounts for 10–15% of all breast cancers [45] and is the most aggressive form of breast cancer with a poor prognosis [46, 47]. Due to a lack of hormone receptors, TNBC patients do not benefit from endocrine therapy. The long-term outcome of chemotherapy in TNBC is inferior due to high rates of relapse and disease recurrence [48, 49]. Thus, new therapeutic strategies to treat TNBC are required. Our lab has extensively worked on the aspect of developing.

DNA methylation, an integral part of the epigenetic machinery, regulates gene expression. Cancer cells utilise DNA methylation as a strategy to overexpress oncogenes, silence TSG, and expression of other regulatory genes [50]. DNA methylation inhibition by drugs like 5-azacytidine(5-AZA) or its deoxy derivative decitabine can lead to hypomethylation, thereby reactivating the expression of silenced, hypermethylated genes and improving patient survival [17] 0.5-AZA, a well known demethylating agent, is a pyrimidine nucleoside analog, also exhibits anticancer activity in haematological malignancies and cytotoxicity in TNBC cells MDA-MB-231 with 83.33 ± 8.82 µM IC50 [51]. A Phase II study treating TNBC with a combination of 5-AZA and entinostat (MS-275) has shown robust efficacy in clinical trials [52]. Another class of drugs known to regulate global DNA methylation are histone deacetylase inhibitors (HDACi). N1-(ferrocenyl)-N8-hydroxyoctanediamide (JAHA), an HDACi, is reported to induce genome-wide DNA hypomethylation at 48 h of exposure to MDA-MB-231 cells [53].

TNBC tumors are characterised by more extensive hypomethylation than hypermethylation [54]. Stirzaker et al. found that TNBC has a distinct methylation pattern that can be stratified to predict survival with prognosis [55]. Interestingly, promoter methylation for genes like BRCA1 [56] and HME1[57] has been reported in TNBC. Here we have used MDA-MB-231, a highly metastatic cell line [47], as a model cell line for TNBC [58] to decipher DNA methylation patterns on ST08 and ST09 treatment. Our previous studies inspired this study - Changes in epitranscriptome(miRNA,mRNA) of curcumin [21], ST08 [22], and ST09 [23] treated breast and ovarian cancer cells.

We performed WGBS after drug treatment to unravel the impact of drugs on global DNA methylation in MDA-MB-231 triple-negative breast cancer cells, correlate the epigenetic changes with gene expression, and define how these regulatory mechanisms impact the expression of specific oncogenes and tumor suppressor genes. A similar study was conducted using 100 µM dose of resveratrol in MDA-MB-231 by Medina-Aguilar et al. [5]and curcumin in colorectal cancer cells by Link et al. [19]. Both studies used an integrative analysis of DNA methylation and gene expression and showed the impact of DNA methylation epigenetic machinery on the expression of oncogenes and TSGs. A mild decrease in hypermethylation to 64% of CpGs in promoters and an increase in hypomethylation to 35% was observed post ST08 and ST09 treatment, indicating that ST08 and ST09 did not induce widespread non-specific global methylation but induced hypomethylation of only a subset of genes. A similar and limited effect in DNA methylation was reported for curcumin in colorectal cancer cells [19] and resveratrol in MDA-MB-231 [5]. We also analysed the gene bodies for differential methylation. 910 and 952 genes in gene bodies were hypermethylated post ST08 and ST09 treatment in MDA-MB-231 cells, respectively. The correlation of the gene body hypermethylation with gene expression revealed CACNAH1 to be upregulated in ST08 treatment and CDH23 upregulation in ST09.CACNA1H is a voltage-gated calcium channel whose expression was found to correlate positively with a decrease in brain metastasis in the breast cancer PDX model [36], and mutations in CDH23 have been found in younger breast cancer patients [37]. Gene body hypermethylation has been reported in Hepatocellular carcinoma [59]. In HCT116 cells treated with 5-Azacytidine-2deoxycytidine, gene body analysis showed that hypermethylation of the gene body was observed upon removal of the drug relatively faster than promoter and correlated with the overexpression of the gene expression [60].

ST08 and ST09 treatment generated a similar altered methylation pattern at CpGisland on chromosome 9. The drug treatment reversed the methylation pattern *CARD9* and *NELFB* seen in breast tumor samples. Pradhan et al. observed hypermethylation of *NELFB/COBRA1* (negative elongation factor/co-factor of BRCA1) in acral melanoma patients, which correlated with worse overall survival [61]. Thus, ST08, ST09 mediated hypomethylation of NELFB predicts a positive outcome. By integrating mRNA expression data, we functionally characterised the CpG island-associated genes on chromosome 9. This analysis showed an exact correlation between *ANKRD18B* gene methylation and expression. Specific Cs in the CpGisland were hypermethylated in treated cells compared to the control, and *ANRKD18* mRNA was downregulated. GEPIA analysis showed *ANKRD18B* mRNA upregulation in tumor samples and associated with low survival in Her2 + ve breast cancer. *ANKRD18B* acts as a tumor suppressor [62]. Thus, *ANKRD18B* has an oncogenic role in breast cancer, and ST08 and ST09 regulate its expression via methylation. Another exciting gene whose expression correlated with methylation was *HOXA10*. *HOXA10*, a developmental control gene, has a tumour-suppressive role in breast cancer [63]. *HOXA10* activates p53 and reduces breast cancer cell invasiveness [64]. ST08 and ST09 induced hypomethylation of *HOXA10*, which correlated with its high expression, indicating a positive outcome.

Interestingly, ST08 and ST09 induced changes in promoter methylation of oncogenes and tumor suppressor genes associated with cellular pathways frequently deregulated in cancer. Another study by Naselli et al. evaluated the effects of indicaxanthin(Ind), a betalain pigment, on DNA methylation and found that Ind induced demethylation in the promoters of some methylation-silenced onco-suppressor genes involved in colorectal carcinogenesis [65].ST08 led to hypermethylation of oncogenes like *IL1B and TMPRSS2*, leading to their downregulation and hypomethylation of TSGs *CDH13 and SYK*, leading to their upregulation. IL1B overexpression is associated with cancer development, metastasis, and poor prognosis in TNBC [66]. *TMPRSS2*, a membrane-bound serine protease, is known to overexpress in the early stages of cancer and increase the severity of pain in these patients. Also, it is known for signal transduction between ECM and cancer cells by activating PAR2 [67] and hence is a novel target in cancer therapeutics [68]. However, promoter analysis of *TMPRSS2* and *SYK* using SMART App showed methylation of these promoters in breast tumor patient samples. SMART App has breast cancer samples of all subtypes, of which TNBC represents the smallest subtype.ST08 hypomethylated promoter of *CDH13* leading to its upregulation.*CDH13* is a known tumor suppressor gene, and its promoter is methylated in breast cancer patients [69, 70] and other cancers as well [71, 72]. Coincidentally, *TET2* transcript levels were upregulated, which might have a role to play in the coactivation of gene expression through the demethylation of enhancers [73]. *TET2* can convert 5-methylcytosine (5mC) to 5-hydroxymethylcytosine (5hmC) and promotes site-specific DNA demethylation [74].

Comparing genome-wide DNA methylation with transcriptomics, we can co-relate ST09 mediated downregulation of oncogenes *SCN5A, RPS6KA2* to promoter hypermethylation, and ST09 mediated upregulation of TSGs *FAT3, PTPN11* to promoter hypomethylation *FAT3* is a known tumor suppressor and is detected as a mutational cancer driver in Breast adenocarcinoma [75]. *SCN5A* gene encodes voltage-gated sodium channels aberrantly expressed in breast cancer and promotes EMT and invasiveness [76]. *RPS6KA2* is overexpressed upon *PI3K* inhibition leading to resistance to PI3K inhibitor treatment [77]. Thus, RPS6KA2 inhibitor and PI3K inhibitor combination therapy are recommended for breast cancer patients with activated RSK. Coincidentally, *DNMT1* and *TET1* were upregulated upon ST09 treatment in MDA-MB-231 cells, suggesting their role in hyper [78, 79] and hypo [80] methylation of genes, respectively.

We also did pathway analysis of genes regulated by ST08 and ST09 mediated DNA methylation. These genes were involved in molecular pathways in cancer. ST08 upregulated ribosome signaling and downregulated pathway related to ECM. This result correlates with the transcriptome study. To induce synthetic lethality, the ST08 mediated upregulated ribosomal signaling can be targeted using Homoharringtonine (HHT), a direct ribosome inhibitor currently used in clinics for cancer treatments [81]. ST09 downregulated the cell-cell signaling pathway and upregulated the DNA double double-strand break process. Cell-cell signaling pathways were enriched in the transcriptome study of ST09 treated MDA-MB-231 cells; however, DNA repair pathways were not enriched. The possible reason behind this is that the genes in this pathway have lower fold change value and high *P*-value; hence they do not fall under significant DE genes. However, WGBS analysis showed that DNA methylation has a role in modulating DNA repair pathways. The DNA repair pathways upregulated via ST09 can be targeted using PARP inhibitors like Olaparib to induce synthetic lethality [82].

## Conclusions

Though DNA methylation has a considerable role in the progression of TNBC, many challenges need to be addressed to apply demethylating agents in the clinic successfully. ST08 and ST09 can modulate gene expression and exert antiproliferative, migrastatic activities based on modifying the epigenome via DNA methylation, suggesting that they may be helpful as a novel epigenetic therapeutic tool. The study thus provides additional mechanistic insights into the potent chemopreventive effect of these novel curcumin derivatives.

## Supplementary Information

**Additional file 1:** **Supplementary Figure 1.** % Distribution ofC(Cytosine) and in combination with other nitrogen bases in control andtreatment samples(ST08,ST09).

**Additional file 2.** List of genes up/down regulated and having hypo/hyper-methylated promoters post ST08 and ST09 treatment.

## Data Availability

The WGBS data is available at PRJNA794262.
