# Supplementary material for: Genome-wide differential DNA methylation analysis of MDA-MB-231 breast cancer cells treated with curcumin derivatives, ST08 and ST09
Source: BMC Genomics. 2022 Dec 6;23:807. doi: 10.1186/s12864-022-09041-2 (PMC9727864; doi:10.1186/s12864-022-09041-2)
Supplement: Supplementary file 1 — Additional file 1: Supplementary Figure 1. % Distribution ofC(Cytosine) and in combination with other nitrogen bases in control andtreatment samples(ST08,ST09). [file 12864_2022_9041_MOESM1_ESM.docx]

Supplementary Figure 1: % Distribution of C(Cytosine) and in combination with other nitrogen bases in control and treatment samples(ST08,ST09).
